# Difficult-to-Treat Rejections in Kidney Transplant Recipients: Our Experience with Everolimus-Based Quadruple Maintenance Therapy

**DOI:** 10.3390/jcm12206667

**Published:** 2023-10-21

**Authors:** Pierre Larsson, Bodil Englund, Jana Ekberg, Marie Felldin, Verena Broecker, Lars Mjörnstedt, Seema Baid-Agrawal

**Affiliations:** 1 Transplantation Center, Sahlgrenska University Hospital, University of Gothenburg, 41345 Gothenburg, Sweden; pierre.larsson@vgregion.se (P.L.); jana.ekberg@vgregion.se (J.E.);; 2 Department of Pathology, Sahlgrenska University Hospital, 41345 Gothenburg, Sweden; 3 Department of Nephrology, Danderyd Hospital, Karolinska Institute, 18288 Stockholm, Sweden

**Keywords:** kidney transplantation, difficult-to-treat rejection, T cell-mediated rejection, chronic antibody-mediated rejection, quadruple therapy, everolimus, graft survival

## Abstract

All chronic and treatment-resistant acute rejections are “difficult-to-treat” and lead to progressive loss of graft function in kidney transplant recipients (KTR), as no effective treatment exists for such rejections to date. We review our experience with a novel strategy to treat such rejections by adding everolimus as a “rescue” to conventional triple maintenance therapy with prednisolone, mycophenolate mofetil and calcineurin inhibitor. We retrospectively analysed data in 28 KTR who received everolimus-based quadruple therapy at our institution for biopsy-proven chronic active T cell-mediated or antibody-mediated rejection (n = 19) or treatment-resistant acute rejections (n = 9) between 2011–2017. The primary outcome was 5-year death-censored graft survival. Main secondary outcomes were response to treatment defined by stable or improved graft function, 5-year patient survival and discontinuation rate of treatment. The Kaplan–Meier estimate for 5-year death-censored graft survival was 79% in all patients, 90% for patients with chronic active T cell-mediated rejections, 78% for chronic active antibody-mediated rejection and 67% for acute rejections. Response to treatment was achieved in 43% and 5-year patient survival was 94%. Treatment was stopped in 12 (43%) patients due to adverse events. Everolimus-based maintenance quadruple therapy, despite high rate of everolimus discontinuation due to adverse events, may be a valid approach in a subset of kidney transplant recipients with such difficult-to-treat rejections, which otherwise would lead to a high rate of graft loss.

## 1. Introduction

Despite significant advances in immunosuppressive therapy in kidney transplant recipients (KTR), some acute rejections are resistant to the available anti-rejection treatments. Steroid-resistant acute T cell-mediated rejections (aTCMR) are traditionally treated with anti-thymocyte globulin (ATG) [1,2] with a success rate of 60–70% [3]; however, ATG may be contraindicated in some patients. No effective treatment exists to date for such aTCMR. Active antibody-mediated rejections (aABMR) are treated extensively with repeated plasmapheresis, high-dose intravenous immunoglobulins (IVIG) and B-cell depleting agents [3]. Despite having received intensive therapy and multiple plasmapheresis treatments, these patients are at high risk for the development of chronic active antibody-mediated rejection (caABMR) and accelerated graft loss. Chronic active T cell-mediated rejections (caTCMR) and caABMR are difficult-to-treat and usually irreversible in nature [4]. Currently, optimizing baseline immunosuppression with steroids + calcineurin inhibitor (CNI) + anti-proliferative agent mycophenolate acid (MPA) is considered the standard of care for patients with chronic rejections. The refractory aTCMR, aABMR as well as all chronic rejections are associated with progressive loss of graft function and eventual graft failure in KTR. No optimal treatment exists for such rejections to date, especially for chronic rejections [5,6].

Everolimus is a mammalian target of rapamycin (mTOR) inhibitor and its potent immunosuppressive action is mediated by impairment of dendritic cell maturation and function, and inhibition of T-cell proliferation [7]. Everolimus is used for the prophylaxis of graft rejection in solid organ transplant recipients [8]. It has a more divergent mode of action than that of CNIs and has been shown to be a potent inhibitor of antibody-mediated immunity [9]. Moreover, it also has antiviral as well as anticarcinogenic properties and is licensed for the treatment of several solid cancers [8,10,11]. Everolimus has proven to be a good substitute for other immunosuppressive drugs in triple maintenance combination therapies in regard to efficacy and safety [12,13,14,15,16,17,18]. Everolimus is known to have synergistic immunosuppressive activity with CNI and has been used in CNI minimization or elimination strategies to reduce CNI exposure and improve graft function after kidney transplantation, mostly in low-risk populations without any rejection [12,13,19,20,21,22]. Efficacy and safety of a quadruple regimen with low doses of both tacrolimus and everolimus has been shown even in lung transplant recipients at five years in a randomized controlled trial [23]. Addition of low-dose everolimus to the conventional triple immunosuppressive therapy, consisting of steroids + CNI + MPA, i.e., quadruple maintenance therapy, may allow CNI minimization, achieving a synergistic immunosuppressive effect that is less nephrotoxic and sufficiently potent to treat difficult rejections in kidney transplant recipients.

We have used this strategy with quadruple maintenance treatment as “rescue therapy” to treat difficult-to-treat acute as well as chronic rejections in KTR at Sahlgrenska University Hospital (SU), Gothenburg, Sweden, since 2011 to enhance overall immunosuppression while reducing toxicity by using lower individual dosage of both CNI and everolimus. We share our experience with this regimen here with a focus on its efficacy and safety.

## 2. Materials and Methods

### 2.1. Study Design and Patients

This retrospective study analysed all KTR who had a diagnosis of biopsy-proven treatment-resistant acute and/or chronic rejections between 2011 and 2017 and received quadruple maintenance immunosuppressive treatment at our centre. The follow-up was performed up to July 2021. Treatment-resistant acute rejections were those that failed to respond, i.e., when serum creatinine failed to return to within 25% of the baseline value within 1 week of completion of treatment and/or had persistent changes of rejection in repeat biopsy after conventional anti-rejection therapy.

### 2.2. Data Collection

Patient demographics, pre-transplant characteristics, post-transplant clinical course, lab values and immunosuppression data were collected using electronic charts and the local electronic registry. Missing values from patients living outside the region were obtained using a national registry. All biopsies after transplantation to the last follow-up were collected using electronic pathology registry. All index biopsies before treatment start were re-evaluated by a renal pathologist in a blinded fashion and diagnosed according to the latest Banff-2019 classification, and cases were categorized as aABMR, aTCMR, caTCMR or caABMR [24].

HLA-antibody analyses were performed using LABScreen^®^Single Antigen assay (One Lambda Inc., Canoga Park, CA, USA), according to the manufacturer’s instructions. Test results were analysed using HLA Fusion 2.0.0 software (One Lambda Inc., Los Angeles, CA, USA). A positive result was defined as a mean fluorescence intensity (MFI) of ≥1000. Donor specific antibodies (DSA) were defined at the antigenic level. In cases where the donor was not HLA typed on all loci, non-typed loci, when possible, were inferred by genetic linkage to typed loci. All DSA-positive (DSA+) patients were identified and the cumulative MFI (cMFI) of each DSA+ test was collected from the local immunology lab database.

### 2.3. Treatment

**Figure 1:**
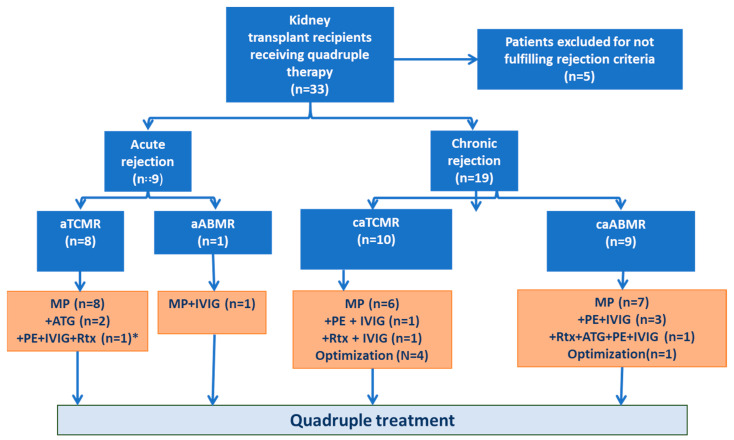
Flow diagram of patients included in the study, their classification according to the type of rejection and the anti-rejection treatment received within 3 months prior to the start of quadruple therapy. * Classified as aAMR at the time of biopsy, re-classified as aTCMR according to Banff-2019 classification. aTCMR, acute T cell-mediated rejection; aABMR, acute antibody-mediated rejection; caTCMR, chronic active T cell-mediated rejection; caABMR, chronic active antibody-mediated rejection; MP, methylprednisolone; ATG, anti-thymocyte globulin; IVIG, intravenous immunoglobulin; PE, plasmapheresis; Rtx, Rituximab.

As shown in Figure 1, aTCMR in KTR was treated first with conventional intravenous (iv) methyl-prednisolone bolus at a dose of 500 mg for 3 days. Steroid-resistant aTCMR was treated with anti-thymocyte globulin (ATG) if not contraindicated, the total dose being 2–6 mg/kg iv divided over 3–6 days. Active ABMR (aABMR) was treated with iv methyl-prednisolone, iv immunoglobulin (IVIG) ±iv anti-CD20 monoclonal antibody rituximab ± plasmapheresis. The doses used were as follows: IVIG for a total dose of 2 g/kg over 5–10 days, plasmapheresis for 5–6 treatments within 5–10 days and rituximab as a single iv infusion at 375 mg/m^2^ body surface area. For acute rejections, if there was a contraindication to ATG in cases of steroid-resistant aTCMR, or the creatinine failed to return to within 25% of the baseline value or signs of rejection persisted on repeat biopsy even after the treatment outlined above for aTCMR or aABMR, a quadruple maintenance therapy with addition of everolimus together with slight reduction in the dose of CNI was commenced in these patients. For chronic rejection, if iv methyl-prednisolone/high-dose oral prednisolone with optimization of immunosuppression did not lead to stabilization or improvement in graft function, then quadruple maintenance treatment was introduced. The initial dose of everolimus was 0.5 mg twice daily and thereafter adjusted to reach trough levels of 3–5 ng/mL. At the same time, the tacrolimus dose was decreased to maintain trough levels of approximately 5 ± 1 ng/mL, with the cumulative everolimus and tacrolimus trough levels targeted to a range of 8–10 ng/mL.

### 2.4. Outcomes

The primary outcome was the 5-year death-censored graft survival after start of treatment. Death-censored graft loss was defined as return to dialysis or re-transplantation. The secondary outcomes were patient survival, safety, rate of discontinuation of treatment and changes in albuminuria and hyperlipidemia (as everolimus may adversely affect these parameters) and patients’ response to treatment assessed by the estimated glomerular filtration rate (eGFR) slope/year from start of treatment to the end of the treatment, calculated using the Chronic Kidney Disease Epidemiology Collaboration (CKD-EPI) Creatinine Equation [25]. The eGFR was calculated at start of treatment, 3 months, 6 months and then yearly until the last follow-up. The patients’ response to treatment was categorized into stable, improved or decreased graft function. An improvement was defined as an eGFR slope of >1 mL/min/1.73 m^2^/year, a decrease was defined as an eGFR slope of <−1 mL/min/1.73 m^2^/year and a stabilisation was defined as the range in between those two [26]. Since the graft function of a patient with refractory rejection is expected to decline with time, a stable function was also considered as a positive result. Thus, response to treatment was defined if the eGFR slope was stable or improved, i.e., the eGFR slope was ≥−1 mL/min/1.73 m^2^/year at the end of treatment from start of treatment.

Regarding safety, emphasis was placed on incidence of infections requiring medical intervention and blood and lymphatic system disorders, which were analysed during the treatment period, and malignancies, which were assessed from the start of the treatment until the most recent follow-up available. Data on infections were collected directly from the electronic medical charts, excluding cytomegalovirus (CMV) and BK virus (BKV), where an event was defined as a clinically symptomatic CMV infection or BKV infection (defined as BKV titers in Geq/mL 10 log > 3), as reported in the medical charts. Blood and lymphatic system disorders included anaemia, thrombocytopenia and leukopenia; each one of which was defined as any event with values below the reference interval from the laboratory performing the analysis and lasting for at least one week. If a value was abnormal at the start of treatment, the first event was counted after the serum level had first returned to normal. If the serum level did not return to normal at the end of treatment, it was defined as persistent. Data on malignancies were collected until the last follow-up, even after discontinuation of the quadruple therapy.

Information on discontinuation of the quadruple therapy regarding the date of discontinuation and the reason for the same was collected by reviewing the medical charts. Albuminuria values were collected at 0, 3, 6 months and then yearly until the last follow-up. The values of cholesterol and triglycerides were analysed at start of treatment, 3 months and then at the end of the treatment. The use of statins at the start of the treatment and any initiation during the treatment was also registered.

### 2.5. Subgroups

All patients were divided into either an acute or a chronic subgroup based on their biopsy-verified rejection type at the time of treatment start. Patients with combined acute and chronic rejection components were placed into the chronic group because of the irreversible nature of the chronic state. The chronic subgroup was further subdivided into caTCMR and caABMR. Comparisons were made between the acute and chronic subgroups and also between acute, caTCMR and caABMR subgroups for all variables and analyses.

### 2.6. Statistical Analysis

Data were analysed using SPSS for windows release 26.0 (SPSS Inc., Chicago, IL, USA). Results of continuous measured data are presented as means ± standard deviation if not stated otherwise. For continuous variables, a paired two sample t-test for means was used when analysing all patients and an unpaired two-sample t-test assuming unequal variance was used when comparing the two subgroups. Fisher’s Exact Test was used for comparing categorical variables. One-way ANOVA and Chi-square tests were used when comparing acute, caABMR and caTCMR subgroups. Patient and graft survival were analysed using the Kaplan–Meier curves with a log-rank test. A two-sided value of *p* < 0.05 was considered statistically significant.

## 3. Results

### 3.1. Patient Disposition and Immunosuppression

In total, 33 patients who received quadruple therapy for treatment-resistant acute or chronic rejection between 2011–2017 based on the biopsy diagnoses according to the valid Banff-classification at the time were identified. Based on a critical and blinded re-evaluation of these biopsies by the pathologist according to Banff-2019 classification, five patients were found not to fulfil all the diagnostic criteria for rejection diagnosis and were therefore excluded and the remaining 28 patients were included in the current analysis. The demographics, pre-transplant and baseline characteristics of the 28 patients are summarised in Table 1 and the anti-rejection treatment received within three months prior to the start of quadruple therapy in Figure 1. Five patients in the acute rejection subgroup could not receive ATG because of contraindications such as CMV or BK viremia, other severe infection, leukopenia, patient frailty or having received thymoglobulin in the past. The median time from Tx to the start of quadruple treatment was 10.5 months (range 1–87 months). The median exposure to the treatment was 36 months (range 3–117 months) and the median follow-up time after initiation of the quadruple therapy was 80.5 months (range 47–121) in all patients. Two patients were between 14–17 years of age at the time of treatment start, all others were adults. All patients were hepatitis B- and C-negative. Two patients were Epstein Barr virus-negative and six had negative CMV serology before Tx. In four patients, quadruple treatment was temporarily stopped due to adverse events (AE) (n = 3) and pregnancy desire (n = 1) before starting again (with duration until resuming therapy again of 2, 3, 3 and 8 months, respectively). The relevant clinical characteristics at start of treatment and until the end of treatment stratified by acute and chronic subgroups are shown in Table 2.

### 3.2. Histopathology and DSA

Nine patients had biopsy-confirmed acute rejection at the start of intervention (8 aTCMR and 1 aABMR). Among the 8 aTCMR, 5 were grade IA, 1 IIA, 1 IB and 1 IIB. Of the 19 patients with chronic rejection, 9 had caABMR (all DSA+) and the remaining 10 had caTCMR (Figure 1). All 10 patients with caTCMR were grade II. Five patients in the caABMR group also had a component of caTCMR grade II. The pathological features including Banff scoring for the chronic subgroups and DSA data are summarized in Table 3.

### 3.3. Primary Outcome (Death-Censored Graft Survival)

**Figure 2:**
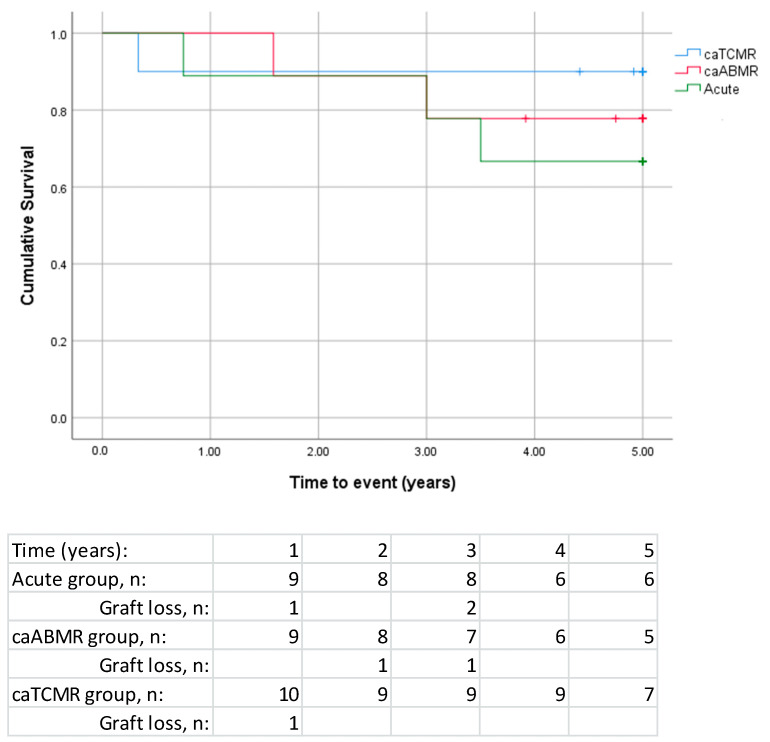
5-year death-censored graft survival in different subgroups: acute rejection (n = 9, including 8 with aTCMR and 1 with aABMR), caABMR (n = 9) and caTCMR (n = 10). The table below the graph shows the number of patients and graft losses for each year. Log-rank *p*-value = 0.53.

In total, 10 grafts were lost during the entire follow-up period and 4 were lost during the treatment period. The death-censored graft survival for all patients at five years was 79%: 90% for patients with caTCMR, 78% for caABMR and 67% for acute rejection (Figure 2). The patients experiencing graft loss during the treatment period had a mean eGFR of 31.0 ± 8.8 at start of treatment whereas those who did not lose their graft had a mean eGFR of 42.9 ± 18.9. The overall graft survival (not censored for deaths) at five years was 74% for all patients, 90% for caTCMR, 62% for patients with caABMR and 67% for the acute rejection subgroup.

### 3.4. Secondary Outcomes

#### 3.4.1. Response to Treatment

In the entire cohort of 28 patients, the mean eGFR (±SD) at start of treatment was 41.2 ± 18.2 mL/min/1.73 m^2^ which declined to 40.6 ± 15.0 mL/min/1.73 m^2^ at one year and 34.6 ± 17.2 mL/min/1.73 m^2^ (n = 8) at five years after start of treatment. The mean eGFR values in acute, caTCMR and caABMR groups are shown in Supplementary Figure S1. The mean eGFR values in individual patients in each group are shown in Supplementary Figure S2. Response to treatment as assessed with eGFR slope/year was observed in 43% (12/28) of all patients at the end of treatment. Five patients improved their eGFR, seven were stable and a decrease was experienced by 16/28 (57%) of all patients. In patients who had DSA at the start of treatment (n = 11), five (46%) responded to treatment, and in patients who were DSA-negative (n = 17), three (18%) responded to treatment. The responders had significantly longer duration of treatment (Table 4). The responders tended to be slightly older and more responders tended to have continued the treatment as compared to the non-responders. Other characteristics were not statistically significant in the two groups. No significant differences were found in the Banff histologic grade or scores between the groups.

#### 3.4.2. Patient Survival

The patient survival was 94% at 5 years for all patients, 80% for caABMR subgroup and 100% for the acute and caTCMR subgroups. In total, five patients died during the median follow up of 80.5 months (all with functioning grafts). Two patients died during the treatment period: one because of cerebral herpes zoster encephalitis (62 months after treatment start) and the other at home (cause of death not known, 82 months after treatment start). The remaining three patients died more than two years after completion of the treatment; the causes of death included pancreatic cancer with liver metastasis 58 months after the stop of treatment (total duration of treatment 3 months) and unspecified in the remaining two.

#### 3.4.3. Safety

The following AE were noted among the patients (Table 5):

1. Infections: Over a cumulative treatment exposure of 1188 months, bacterial infections were the most common infection type with 2 events in 11 patients. Fifteen infection events in 10 patients were considered serious and required hospitalization. Among the 14 events of sepsis, one patient alone had 6 episodes. There were 20 events of viral infection in 20 patients and 3 were considered serious and required hospitalization;
2. Hematologic and lymphatic disorders: Over a cumulative treatment exposure of 1188 months, the most common haematological disorder was anaemia. A total of 4 patients had a haemoglobin value below normal from study start until the end and 25 patients developed anaemia short- or long-term during the follow up. As regards leukopenia, there was only one patient with persistent leukopenia and in total 15 events in nine patients. Out of the 24 events in the 11 patients with thrombocytopenia, 5 had persistent thrombocytopenia. Six patients had serious cytopenias, of which two were admitted for severe leukopenia, and the other four received 1–3 units of blood for severe anaemia;
3. Malignancies: Over a cumulative follow-up of 2262 months (up to the last follow-up or death, which included period even after stopping the treatment), twelve patients (43%) developed a malignancy at some point after having started quadruple treatment (Table 5). Two patients developed serious malignancies, of which both were fatal—one developed amelanotic melanoma and pancreatic cancer, and the other one developed lymph node metastasis from a carcinoma (possibly renal origin). The patient with amelanotic melanoma and pancreatic cancer was only on treatment for three months, developed the malignancies 48 months after discontinuation of treatment and died later from liver metastasis. The other patient was diagnosed with lymph node metastasis from a carcinoma 81 months after discontinuation and later died.

#### 3.4.4. Discontinuation of Quadruple Treatment

In 12/28 patients (43%), the quadruple treatment (everolimus in all and MMF as well in 2 patients) had to be discontinued because of AE [leukopenia, n = 1; albuminuria, n = 1; chronic norovirus infection, n = 1; deep vein thrombosis/pulmonary embolism, n = 1; vomiting/nausea, n = 1; multiple infections, n = 1; interstitial lung disease, n = 1; elevated transaminases, n = 1; low-grade rectal cancer, n = 1; fulminant nephrotic syndrome, n = 1; tiredness, n = 1; varicella zoster virus encephalitis, n = 1]. The median duration of treatment before terminating treatment due to AE was 27 (range 3–87) months. Three of these 12 patients improved their eGFR slope and two were stable compared to start of treatment while on treatment. No Significant differences were found when comparing patients who continued treatment compared to those who stopped treatment (Supplementary Table S1).

#### 3.4.5. Albuminuria

Only 19/28 (68%) patients had data for albuminuria available before and after quadruple treatment, as measured with a urine albumin/creatinine ratio (UACR) (Table 3). No significant difference was found between start of treatment and end of treatment values. However, treatment had to be discontinued in one patient due to significant albuminuria after the start of treatment.

#### 3.4.6. Lipid Profile

In total, 17 and 16 patients had measurements available for cholesterol and triglycerides, respectively (Table 3). No significant difference was found between start of treatment and end of treatment values. However, the number of patients that were receiving lipid-lowering drugs at the start of intervention was nine (32%) and an additional nine patients (32%) initiated lipid-lowering treatment during the intervention period.

### 3.5. Comparison of Subgroups

When comparing the acute and chronic subgroups, no significant differences in death-censored/overall graft and patient survival, response rate, as well as AE were found (Figure 2 and Table 3 and Table 5). The number of patients who responded to treatment as assessed with the annual eGFR slope was five (50%) in the caTCMR group, three (33%) in the caABMR group and four (44%) in the acute group (*p* = NS). When analysing the caTCMR, caABMR and acute subgroups, mean eGFR, and DSA-positivity at start of treatment were significantly higher in the caABMR subgroup as compared to the other subgroups (Table 6 and Figure S1). 

## 4. Discussion

In this study, we report in 28 kidney transplant recipients the long-term clinical outcomes of the use of everolimus-based quadruple immunosuppressive regimen for treatment-resistant acute rejections and/or chronic rejections, both of which are difficult-to-treat. The 5-year death-censored graft survival for all patients was 79%: 90% for patients with caTCMR, 78% for caABMR and 67% for acute rejection subgroups. In total, 43% of patients responded to the treatment as determined by a stable or improved annual eGFR slope. The number of responders in the caTCMR, caABMR and acute rejection subgroups were 50%, 33% and 44%, respectively. However, the difference was not statistically significant.

Notably, the 5-year death-censored graft survival of 78% observed in the caABMR subgroup (n = 9) in our cohort is significantly better as compared to that reported in other studies. Redfield et al. have shown in a larger cohort of patients (n = 123) with caABMR (with both presence of DSA and C4d and/or microvascular inflammation apart from chronic changes in biopsy) a median graft survival of only 1.9 years and even worse if no anti-rejection treatment with steroids and IVIG was given [27]. Another multicentre study that included 91 patients with caABMR observed death-censored graft survival of 36.4% at 5 years [28]. Other studies in patients with caABMR have observed a graft survival of 50% at 1–2 years and 32% at five years after diagnosis [5,27,29]. DSA+ KTR have been proven to have a higher risk of graft loss [30,31]; however, all our patients in the caABMR group had DSA. Moreover, all the patients in our cohort had a cg score ≥ 1 and ifta ≥ 30%; 67% (6 of 9) had a g+ptc score ≥ 3, 44% (5 of 9) had I + t scores ≥ 3 and 56% (5 of 9) were C4d positive (on paraffin). Thus, the severity of the caABMR phenotype in our cohort appears at least not milder than that reported in the previous studies. Our results suggest that everolimus-based quadruple therapy may be an option also for patients with caABMR. However, because of the small numbers and retrospective nature of the study, more data are needed to corroborate our findings. Regarding caTCMR, the data on graft survival are sparse since the histologic diagnosis of caTCMR has been incorporated into the Banff classification only in 2017 and further clarified in 2019 [24,32]. In a previous study that classified all rejections according to Banff 2013 classification, the graft survival at 21 months after biopsy-proven diagnosis of any TCMR, where probably both acute and chronic TCMR were grouped together, was reported to be 54%, which appears to be significantly worse than the 90% found in our study for caTCMR alone at five years [33]. Pertaining to steroid-resistant aTCMR, the 5-year graft survival rate of 67% observed in our study is slightly lower than the 70-80% reported in several past studies where thymoglobulin was used for steroid-resistant rejections [34,35,36]. However, the majority (63%) of our aTCMR patients included here had a contraindication for the use of thymoglobulin, which may explain the slightly lower rates observed in our study.

The treatment with everolimus-based quadruple therapy was well-tolerated by approximately 57% patients in our study with treatment discontinuations due to AEs in the remaining 43% after a median duration of 27 (3–87) months. A randomized study comparing everolimus conversion approximately 7 years after transplantation versus cyclosporine continuation as maintenance therapy also showed high rate of discontinuation in the everolimus-arm (32.6%) after one year [37]. On the other hand, another randomized study with CNI minimization and conversion to everolimus in stable kidney transplant recipients 5.6 years after transplantation showed a discontinuation rate of only 16.7% [20]. However, our patient population was much more immunosuppressed owing to several, and more intensive, anti-rejection treatments received in the past for their difficult-to-treat rejections, which may explain the higher discontinuation rate in our study. The AE profile observed in this study also appears similar to those in other studies [27,31,37,38]. A retrospective study comparing conversion to either mTORi or belatacept in 79 difficult-to-treat patients on CNI-based or mTORi-based maintenance immunosuppression (mainly because of CNI toxicity or mTOR-inhibitors-induced AEs) had similar outcomes in terms of safety and cumulative survival as our study [38]. Previous studies have also pointed out a higher incidence of anaemia when using everolimus [39].

Two 12-months randomized studies comparing everolimus to CNI regimen as maintenance therapy found a rate of serious infections of 5.8% and 17.4%, respectively [30,37]. Patients receiving quadruple therapy in the current study had a 36% rate of serious infections, but here a less strict definition was used compared to the other two studies and the treatment duration was much longer. The two studies included only fatal and life-threatening incidents, whereas the present study included all those who required hospitalization. Furthermore, our patient population was much more immunosuppressed owing to several and more intensive anti-rejection treatments in the past for their difficult-to-treat rejections. Thus, more data from prospective randomized studies are needed to better assess the safety of the everolimus-based quadruple treatment. Analysis of other major AEs, such as cardiac and gastrointestinal disorders as well as other serious events requiring hospitalisation would also be of value.

Kidney transplant recipients are known to have a higher risk of cancer than the general population, especially skin cancers and Kaposi’s sarcoma [31,40]. Paradoxically, CNIs are associated with an increased risk of cancer, while everolimus has been proven to have antioncogenic effects [8]. In the present study, 24 events of malignancy occurred in 12 patients and 2 patients developed life-threatening malignancies long after discontinuation of quadruple treatment [38]. This increased risk of malignancies may be due to increased overall immunosuppression these patients received due to prior repeated treatments for rejection and not necessarily due to quadruple treatment per se. In a retrospective study analysing a cohort with 3537 Italian KTR receiving various combinations of immunosuppression, 263 (7.5%) patients developed a non-melanoma skin cancer and 253 (7.2%) developed another type of cancer [31]. In the present study, 8/28 (28.6%) developed a non-melanoma skin cancer and 4/28 (14.3%) patients developed another type of cancer. However, the different definitions used in the two studies and different post-transplant factors, such as follow-up time and immunosuppression/anti-rejection treatments received, make the comparison difficult.

The mean albuminuria appeared to increase at the end of treatment, even though not statistically significant. This supports the notion that everolimus is associated with a higher rate of albuminuria, as also described in other studies [39,41]. Albuminuria has been found to be a negative predictor of patient and graft survival, yet the patient and graft survival outcomes in this study were fairly good despite increased albuminuria [42,43,44]. Cardiovascular risk factors, including hyperlipidaemia, have also shown to affect patient and graft survival [45,46]. In the present study, substantial increase in hypercholesterolemia when using everolimus, as reported in other studies, did not seem to occur [39], which could also be explained by the increased use of statins in our patients. However, it should also be admitted that both albuminuria and lipid analyses suffered from missing values (patients with complete data were n = 19 and n = 16, respectively) and therefore, conclusive statements cannot be made.

Our study has several strengths. To our knowledge, this is the first study to evaluate a novel approach with everolimus-based quadruple maintenance therapy in patients with treatment-resistant acute rejections as well as chronic rejections in whom no established therapy exists to date. Furthermore, a comprehensive and thorough investigation of multiple clinical, laboratory and histopathological factors was performed over long-term in these patients. There are also some limitations of the study that need to be mentioned. Some of the limitations are those that are inherently associated with the retrospective analysis study design, the most important of which is lack of a control group for comparison. We acknowledge that it is difficult to draw any conclusions regarding the treatment response and adverse events given the limitations in our study design, as it was not randomized, and as such, subjected to selection bias. Another limitation is the heterogeneous and small sample size which limits the power of the study. Moreover, follow-up biopsies were not available in many of the patients and the data on albuminuria and lipid profile were missing in some patients.

## 5. Conclusions

The results of this study suggest that quadruple maintenance therapy with low-dose everolimus and minimization of CNI may be a viable rescue treatment for a subgroup of patients with difficult-to-treat chronic rejections who would otherwise lose their graft due to lack of further treatment options and those treatment-resistant acute rejections with contraindications to a second line of treatment with thymoglobulin. The responders were found to have significantly longer duration of treatment as compared to non-responders, indicating that those who could tolerate and continue the treatment for a longer duration had beneficial effects. However, discontinuation of treatment in 43% of patients due to AEs is of concern and warrants very careful selection and monitoring of these patients. Randomized controlled trials are needed to further evaluate this regimen in terms of efficacy and safety for which our results may form an important basis.

## Figures and Tables

**Table 1:** Pre-transplant and baseline characteristics *.

Patient Characteristics *	caTCMR (n = 10)	caABMR (n = 9)	Acute Group (n = 9)
Mean age ± SD, years (range)	53.7 ± 14.6 (26–75)	36.6 ± 19.9 (14–70)	45.3 ± 16.0 (20–65)
Males, n (%)	5 (50)	2 (22)	6 (67)
Underlying disease, n (%)			
Unknown	2 (20)	1 (11)	2 (22)
Glomerulonephritis	1 (10)	4 (44)	4 (44)
Polycystic kidney disease	3 (30)	1 (11)	1 (11)
Diabetes	0	0	0
HUS/TTP	2 (20)	0	0
Others **	2 (20)	3 (33)	2 (22)
Median duration of dialysis before Tx (range), months	24 (9–45)	15 (1–47)	20 (4–83)
Patients with >1 kidney Tx, n (%)	5 (50)	2 (22)	3 (33)
DD/LD, n (%)	7/3 (70/30)	6/3 (67/33)	7/2 (77/23)
ABO incompatible, n	1 (10)	0	2 (22)
HLA sensitization >20% (class 1 or 2), n (%)	3 (30)	2 (22)	2 (22)
Median HLA-mismatch grade (range)			
Class 1	2 (0–3)	2 (1–4)	3 (0–4)
Class 2	1 (0–2)	2 (1–2)	2 (0–2)
Median MMF AUC after Tx (range), µg/LxH			
Day 4 (n = 23)	66.5 (45–84)	49.5 (35–63)	52 (40–135)
3 months (n = 20)	41 (24–176)	66 (62–86)	55 (37–112)
Mean time from Tx to start of treatment ± SD, months	19.5 ± 27.2	36.6 ± 25.0	14.7 ± 17.9
Mean duration of treatment ± SD, months	41.4 ± 37.8	45.6 ± 25.8	40.4 ± 30.6
Nr. of patients on CNIs at start of treatment, n (%)			
Tacrolimus	9 (90)	9 (100)	7 (78)
Cyclosporine	1 (10)	0	2 (22)
Patients starting statin treatment, n (%)			
Before/at start of treatment	5 (50)	2 (22)	2 (22)
During quadruple treatment	3 (30)	4 (44)	2 (22)

Abbreviations: Tx, transplantation(s); SD, standard deviation; HLA, human leukocyte antigen; DD, deceased donor; LD, living donor; MMF AUC, Mycophenolate mofetil area under the curve; HUS, hemolytic uremic syndrome; TTP, thrombotic thrombocytopenic purpura. * There were no significant differences between the three subgroups. ** Other causes included interstitial nephritis, hereditary nephropathy, congenital renal hypoplasia (n = 2), renal vascular disease due to hypertension or other causes (n = 2) and multisystem disease.

**Table 2:** Clinical characteristics at start of treatment and during treatment.

Characteristics	Chronic Group (n = 19)				Acute Group (n = 9)			
	Start of Treatment Level	Mean Treatment Level	End of Treatment Level	*p*-Value	Start of Treatment Level	Mean Treatment Level	End of Treatment Level	*p*-Value
Mean creatinine ± SD, µmol/L	160 ± 44	165 ± 45	243 ± 176	0.05	183 ± 53	162 ± 71	253 ± 174	NS
Mean eGFR ± SD, ml/min/1.73 m^2^	44 ± 21	41 ± 6	31 ± 15	<0.01	36 ± 9	47 ± 4	33 ± 19	NS
Mean albuminuria ± SD, g/mol creatinine (n = 19)	31 ± 51	38 ± 20	76 ± 61	0.05	18 ± 20	35 ± 57	80 ± 151	NS
Mean Tacrolimus trough levels ± SD, µg/L (n = 33)	7.6 ± 2.1	5.4 ± 1.4	4.9 ±1.6		7.9 ± 2.5	6.1 ± 1.3	4.7 ± 1.1	
Mean everolimus trough levels ± SD, µg/L (n = 28)	0	3.2 ± 1.2	3.3 ± 1.8		0	3.2 ± 1.4	3.2 ± 1.8	
	Start of treatment level	3 months after conversion	End of treatment level	*p*-value	Start of treatment level	3 months after conversion	End of treatment level	*p*-value
Mean cholesterol ± SD, mmol/L (n = 17)	5.3 ± 2.0	5.3 ± 1.0	5.1 ± 1.2	NS	5.6 ± 1.3	6.1 ± 1.2	7.0 ± 4.8	NS
Mean triglycerides ± SD, mmol/L (n = 16)	2.2 ± 1.9	2.6 ± 1.6	2.0 ± 0.7	NS	1.7 ± 0.3	2.0 ± 0.6	4.4 ± 6.5	NS

Abbreviations: SD, standard deviation; eGFR, estimated glomerular filtration rate; NS, not significant.

**Table 3:** Pathological features and DSA data related to the index biopsies of chronic rejection based on Banff 2019 classification.

Pathological Features	caTCMR (n = 10)	caABMR (n = 9)
Mean percentage of sclerosed glomeruli	6	14
Interstitial fibrosis and tubular atrophy at start of treatment, median percentage	32.5	40
Glomerulitis, n		
g0	5	1
g1	4	2
g2	1	4
g3	0	2
TG, n		
cg0	6	0
cg1a	0	3
cg1b	3	4
cg2	1	2
cg3	0	0
Capillaritis, n		
ptc0	7	3
ptc1	2	2
ptc2	1	4
ptc3	0	0
Tubulitis, n		
t0	0	1
t1	7	8
t2	3	0
t3	0	0
Intersitital inflammation, n		
i0	1	0
i1	6	5
i2	1	3
i3	2	1
Intimal arteritis, n		
v0	7	9
v1	1	0
v2	1	0
v3	0	0
Intimal fibrosis, n		
cv0	0	2
cv1	0	1
cv2	5	4
cv3	5	2
Intimal fibroplasia, n	8	6
Cellular infiltrates in intima, n	10	6
C4d+, n	0	5
DSA+, n	2	9

Abbreviations: caTCMR, chronic active T cell-mediated rejection; caABMR, chronic active antibody-mediated rejection; DSA, donor-specific antibody; TG, transplant glomerulopathy

**Table 4:** Variables comparing patients who responded to treatment versus non-responders.

Variables	Responders (n = 12)	Non-Responders (n = 16)	*p*-Value
Mean age ± SD, years	51.8 ± 14.7	40.8 ± 22.1	0.09
Males, n (%)	5 (42)	8 (50)	0.66
HLA sensitization >20% (class 1 or 2), n (%)	2 (17)	5 (31)	0.38
Median HLA mismatch grade (range)			
class 1	2.5 (0–4)	2.5 (1–4)	0.66
class 2	1.5 (0–2)	1 (0–2)	0.52
Histopathology of index biopsy:			
Acute/Chronic rejection (%)	4/8 (33/67)	5/11 (31/69)	0.90
aTCMR/aABMR (n = 9)	3/1	5/0	
caTCMR/caABMR (n = 19)	5/3	5/6	0.46
Banff grade (aTCMR, n = 8)			
IA	1	4	
1B	0	1	
IIA	1	0	
IIB	1	0	
Mean Banff scores (chronic rejection)			
g	0.8 ± 0.7	1.5 ± 1.1	0.11
ptc	0.5 ± 0.9	0.9 ± 0.8	0.34
t	1 ± 0.5	1.2 ± 0.4	0.43
i	1.5 ± 0.8	1.5 ± 0.9	0.90
v	0.25 ± 0.7	0.1 ± 0.3	0.56
cv	2.4 ± 0.5	1.9 ± 1.1	0.25
TG, n (%)	3 (25)	7 (44)	0.30
C4d+, n (%)	4 (33)	3 (19)	0.38
DSA+ at start of treatment, n (%)	7 (59)	6 (38)	0.27
Mean eGFR at start of treatment ± SD, ml/min/1.73 m^2^	36.8 ± 9.1	44.5 ± 24.6	0.23
Mean albuminuria at start of treatment ± SD, g/mol (n = 19)	29.7 ± 59.0	23.2 ± 20.5	0.72
Mean cholesterol at start of treatment ± SD, mmol/L	6.0 ± 2.1	4.6 ± 1.1	0.12
Median tacrolimus trough level at start of treatment (range), µg/L	7.6 (5.3–13)	6.9 (4.6–10)	0.24
Mean duration of treatment ± SD, months	62.7 ± 31.3	27.3 ± 24.5	<0.01
Mean eGFR after 1 year of treatment ± SD, ml/min/1.73 m^2^	44.8 ± 14.6	38.1 ± 12.7	0.26
Mean albuminuria after 1 year of treatment ± SD, g/mol (n = 19)	21.5 ± 21.9	66.2 ± 60.3	0.08
Median tacrolimus trough level after 1 year of treatment (range), µg/L	4.8 (4.0–9.0)	5.3 (2.6–12.9)	0.52
Median everolimus trough level after 1 year of treatment (range), µg/L	3.3 (2.0–7.6)	3 (2.0–4.5)	0.37
Continuation of treatment, n (%)	5 (42)	2 (13)	0.08
Graft survival (functioning graft), n (%)	9 (75)	8 (50)	0.18
Mortality, n (%)	1 (8)	4 (25)	0.25

Abbreviations: caTCMR, chronic active T cell-mediated rejection; caABMR, chronic active antibody-mediated rejection; HLA, human leukocyte antigen; DSA+, donor-specific antibody positive; TG, transplant glomerulopathy; eGFR, estimated glomerular filtration rate.

**Table 5:** Adverse events during treatment (cumulative exposure = 1188 months) *.

Adverse Events **	Events, n			Number of Events per Month of Exposure		
	All Patients (n = 28)	Chronic Group (n = 19)	Acute Group (n = 9)	All Patients	Chronic Group	Acute Group
Infections and infestations						
Bacterial	23 events in 11 patients	15 events in 9 patients	7 events in 2 patients	0.019	0.018	0.019
Urosepsis	9	7	1			
Pneumonia	2	2	0			
Sepsis	7	1	6			
*Pneumocystis jiroveci*	1	1	0			
Catheter-related infections	2	2	0			
*Salmonella gastroenteritis*	1	1	0			
*Clostridium difficile*	1	1	0			
Viral	20 events in 20 patients	16 events in 15 patients	4 events in 5 patients	0.017	0.019	0.008
Flu	4	4	0			
*Varicella zoster*	2	2	0			
Herpes zoster	3	3	0			
Herpes simplex	1	0	1			
Norovirus	3	3	0			
Cytomegalovirus	2	2	0			
BK virus	5	2	3			
Serious infections requiring hospitalization	15 events in 10 patients	12 events in 7 patients	3 events in 3 patients	0.013	0.015	0.008
Blood & lymphatic system disorders (n = 31)						
Anaemia	55 events in 25 patients	41 events in 18 patients	14 events in 7 patients	0.046	0.050	0.038
Leukopenia	15 events in 9 patients	10 events in 6 patients	5 events in 3 patients	0.013	0.012	0.014
Thrombocytopenia	24 events in 11 patients	17 events in 9 patients	7 events in 2 patients	0.020	0.021	0.019
Serious cytopenias requiring intervention ^Ω^	8 events in 6 patients	5 events in 4 patients	3 events in 2 patients	0.007	0.006	0.008
Malignancy	24 events in 12 patients	16 events in 7 patients	8 events in 5 patients	0.011	0.009	0.009
Non-melanocytic skin cancer	19	12	7			
Others ^Ψ^	5	4	1			
Death ^¥^	5	4	1	0.002	0.002	<0.001

* For malignancies and deaths, total cumulative exposure was 2262 months. ** Defined as events requiring medical intervention (except for cytomegalovirus, BK virus and blood & lymphatic disorders which were based on laboratory values). ^Ω^ Serious cytopenias defined as those requiring discontinuation, hospital admission or transfusion. ^Ψ^ Other malignancy types included pancreatic cancer, atypical lipomatous tumour/well-differentiated liposarcoma, amelanotic melanoma, low-grade rectal adenocarcinoma and lymph node metastasis from a carcinoma (possibly from renal origin). ^¥^ Causes of deaths included pancreatic cancer and cerebral herpes encephalitis. All patients died with functioning grafts and five patients died more than two years after completion of treatment.

**Table 6:** Variables comparing caTCMR, caABMR and acute rejection subgroups.

Variables	caTCMR (n = 10)	caABMR (n = 9)	Acute (n = 9)	*p*-Value
Mean age ± SD, years	54 ± 14.6	36 ± 19.9	45 ± 15.9	0.11
Males, n (%)	5 (50)	2 (22)	6 (67)	0.16
HLA sensitization >20% (class 1 or 2), n (%)	3 (30)	2 (22)	2 (22)	0.90
HLA mismatch grade, median (range)				
class 1	2 (0–3)	2 (1–4)	3 (0–4)	0.21
class 2	1 (0–2)	2 (1–2)	2 (0–2)	0.12
DSA+ at baseline, n (%)	2 (20)	9 (100)	0	<0.01
Mean baseline eGFR ± SD, mL/min/1.73 m²	35 ± 9.1	54 ± 25.6	36 ± 9.2	0.03
Mean baseline albuminuria ± SD, g/mol (n = 24)	25 ± 20.5	38 ± 76.7	18 ± 20.3	0.67
Mean tacrolimus trough level during treatment ± SD, µg/L	5.5 ± 1.3	5.3 ± 1.7	6.1 ± 1.3	0.56
Mean everolimus trough level during treatment ± SD, µg/L	3.2 ± 1.3	3.1 ± 1.2	3.2 ± 1.4	0.99
Mean duration of treatment ± SD, months	41 ± 37.8	46 ± 25.8	40 ± 30.6	0.11
Continuation of treatment, n (%)	6 (60)	5 (56)	5 (56)	0.97
Graft survival (functioning graft), n (%)	9 (90)	8 (89)	7 (78)	0.27
Response to treatment, n (%)	5 (50)	3 (33)	4 (44)	0.76
Patient survival (deaths), n (%)	2 (20)	2 (22)	1 (11)	0.80

Abbreviations: caABMR, chronic active antibody-mediated rejection; caTCMR, chronic active T cell-mediated rejection; eGFR, estimated glomerular filtration rate; DSA+, donor-specific antibody positive; HLA, human leukocyte antigen.

## Data Availability

The data presented in this study are available on request from the corresponding author. The data are not publicly available due to data protection and ethical restrictions.

## Supplementary Materials

The following supporting information can be downloaded at: https://www.mdpi.com/article/10.3390/jcm12206667/s1, Figure S1: Mean eGFR at the start of treatment and the first 5 follow-up years in the subgroup of patients with acute rejection (n = 9, including 8 with aTCMR and 1 with aABMR), caTCMR (n = 10) and caABMR (n = 9). The dotted line shows the mean eGFR in all patients. After graft loss, eGFR was imputed to 10 ml/min/1.73m²; Figure S2: Individual eGFR curves based on rejection subgroups; Table S1: Variables comparing patients who continued versus discontinued treatment.
