# Supplementary material for: Difficult-to-Treat Rejections in Kidney Transplant Recipients: Our Experience with Everolimus-Based Quadruple Maintenance Therapy
Source: J Clin Med. 2023 Oct 21;12(20):6667. doi: 10.3390/jcm12206667 (PMC10607360; doi:10.3390/jcm12206667)
Supplement: Supplementary file 1 [file jcm-12-06667-s001.zip › jcm-2673284-supplementary.pdf]

Supplementary Material

Figure S1

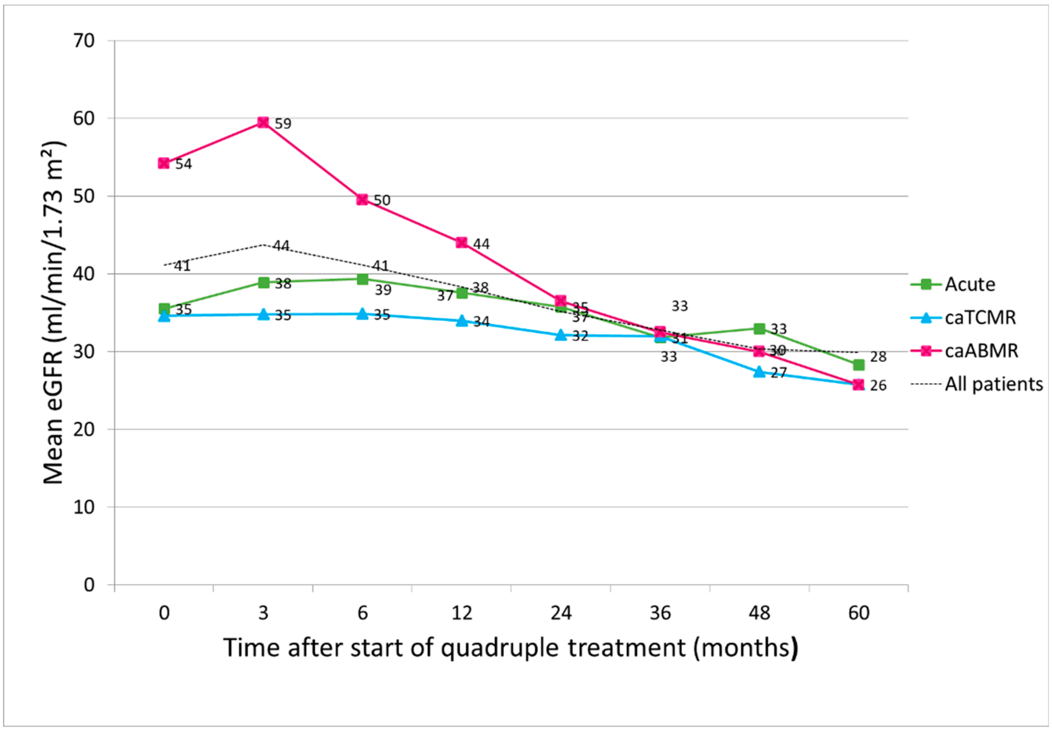

Month:	0	3	6	12	24	36	48	60
A) Acute group, n:	9	9	9	9	8	7	6	3
B) caTCMR, n:	10	10	9	9	8	6	5	5
C) caABMR, n:	9	9	9	9	8	7	6	3

Figure S1. Mean eGFR at the start of treatment and the first 5 follow-up years in the subgroup of patients with acute rejection (n=9, including 8 with aTCMR and 1 with aABMR), caTCMR (n = 10) and caABMR (n = 9). The dotted line shows the mean eGFR in all patients. After graft loss, eGFR was imputed to 10 ml/min/1.73m².

Abbreviations: caTCMR, chronic active T cell-mediated rejection; caABMR, chronic active antibody-mediated rejection; eGFR, estimated glomerular filtration rate.

Figure S2: Individual eGFR curves based on rejection subgroups.

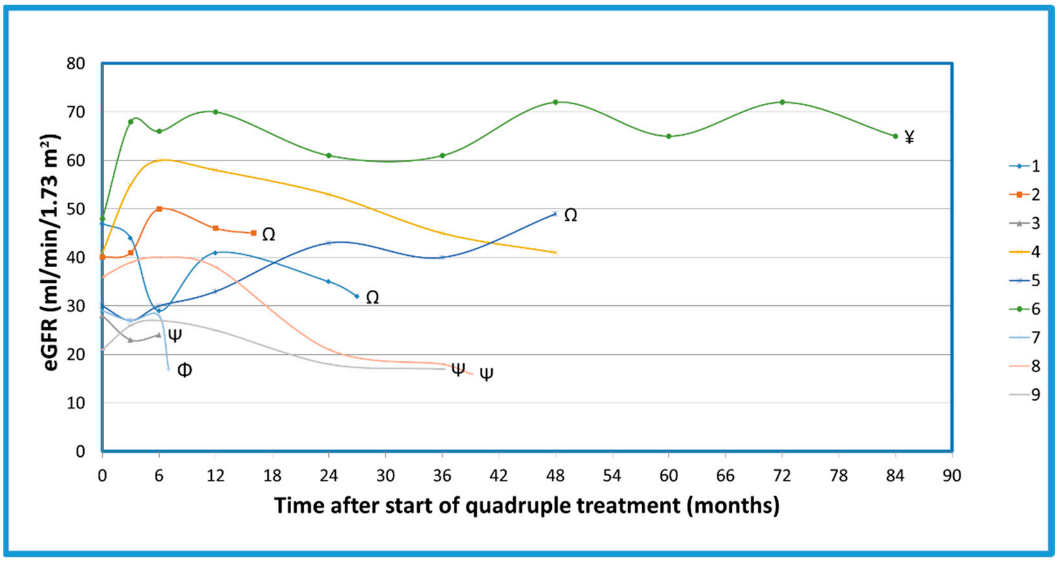

Figure S2a. Individual eGFR curves in patients with acute rejection. Patient nr. 2 was included in the aABMR group and the others were included in the aTCMR group.

Abbreviations: aTCMR, acute T cell-mediated rejection; aABMR, active antibody-mediated rejection; eGFR, estimated glomerular filtration rate.

ΩStopped treatment due to adverse events.
ΨStopped treatment due to graft loss.
¥Stopped treatment due to death.
ΦThe reason for stopping the treatment was not known.
Those with no symbols were still on treatment at the end of follow-up.

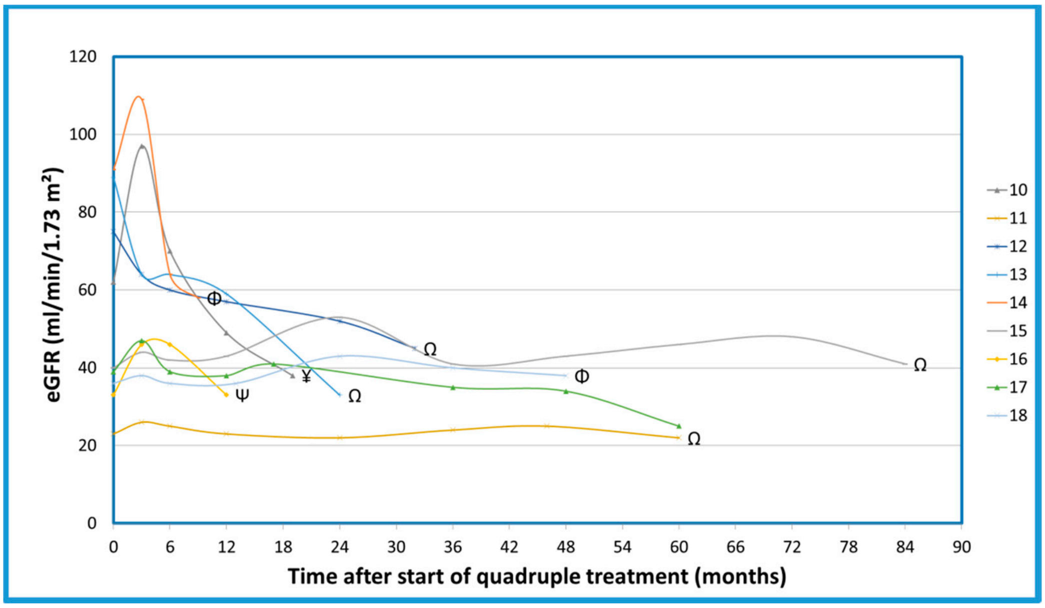

Figure S2b. Individual eGFR curves in patients with caABMR

Abbreviations: caABMR, chronic active antibody-mediated rejection; eGFR, estimated glomerular filtration rate.

ΩStopped treatment due to adverse events.
ΨStopped treatment due to graft loss.
¥Stopped treatment due to death.
ΦThe reason for stopping the treatment was not known.
Those with no symbols were still on treatment at the end of follow-up.

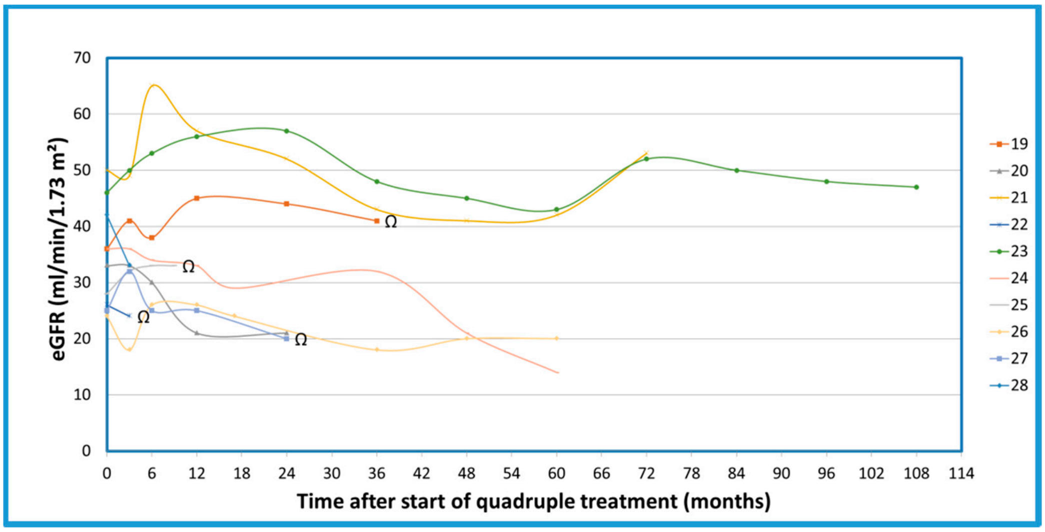

Figure S2c. Individual eGFR curves in patients with caTCMR

Abbreviations: caTCMR, chronic active T cell-mediated rejection; eGFR, estimated glomerular filtration rate.

ΩStopped treatment due to adverse events.
ΨStopped treatment due to graft loss.
¥Stopped treatment due to death.
ΦThe reason for stopping the treatment was not known.
Those with no symbols were still on treatment at the end of follow-up.

Table S1. Variables comparing patients who continued versus discontinued treatment

Variables	Continued (n=16)	Discontinued (n=12)	P-value
Mean age ± SD, years	42.6 ± 17.6	49.4 ± 17.9	0.32
Males, n (%)	7 (44)	6 (50)	0.74
HLA sensitization >20% (class 1 or 2), n (%)	3 (19)	4 (33)	0.38
HLA mismatch grade, median (range)			
class 1	2.5 (0-4)	2.5 (0-3)	0.42
class 2	1.0 (0-2)	1.5 (0-2)	0.53
Acute / Chronic rejection, n (%)	5 / 11 (31 / 69)	4 / 8 (33 / 67)	0.91
caTCMR / caABMR	8 / 3	3 / 5	0.13
DSA+ at start of treatment, n (%)	6 (38)	7 (58)	0.27
C4d+ at start of treatment, n (%)	4 (25)	3 (25)	1
Transplant glomerulopathy at start of treatment, n (%)	5 (31)	5 (42)	0.57
Mean eGFR at start of treatment ± SD, mL/min/1.73 m²	39.4 ± 16.2	43.7 ± 21.1	0.55
Mean eGFR at end of treatment ± SD, mL/min/1.73 m²	29.4 ± 18.7	34.3 ±11.5	0.43
Mean cholesterol at start of treatment ± SD, mmol/L	5.1 ± 1.7	5.7 ± 2.1	0.54
Mean albuminuria at start of treatment ± SD, g/mol (n=24)	26.3 ± 19.3	26.6 ± 65.3	0.99
Mean albuminuria at end of treatment ± SD, g/mol (n=24)	80.2 ± 89.6	70.7 ± 66.9	0.81
Mean duration of treatment ± SD, months	48.6 ± 34.3	34.3 ± 24.8	0.23
Median tacrolimus trough level at start of treatment (range), µg/L	8.1 (5.5-13)	6.8 (4.6-12)	0.17
Median tacrolimus trough level at end of treatment (range), µg/L	4.4 (2.9-8.2)	5.3 (2.6-6.7)	0.92
Responders vs non-responders, yes/no (%)	6 / 10 (37.5 / 62.5)	6 / 6 (50 / 50)	0.51
Graft survival (functioning graft) , n (%)	13 (81)	11 (92)	0.44
Mortality, n (%)	2 (12.5)	3 (25)	0.39

Abbreviations: caTCMR, chronic active T cell-mediated rejection; caABMR, chronic active antibody-mediated rejection; HLA, human leukocyte antigen; DSA+, donor-specific antibody positive; eGFR, estimated glomerular filtration rate.
